# Dissociative symptoms during the SARS-CoV-2 pandemic situation in mental health patients

**DOI:** 10.1186/s40479-026-00363-1

**Published:** 2026-07-27

**Authors:** Max C. Pensel, Johanna M. H. L. Siems, Alexander Blazejak, Charlotte Steinau, Jana Fink, Marie-Therese Schmitz, Alexandra Philipsen, Henrik Rohner

**Affiliations:** 1 Department of Psychiatry and Psychotherapy, University Hospital Bonn, Venusberg-Campus 1, 53127 Bonn, Germany; 2 Department of Epileptology, University Hospital Bonn, Bonn, Germany; 3 Institute for Medical Biometry, Informatics and Epidemiology, University of Bonn, Bonn, Germany

**Keywords:** Dissociation, Depersonalization, Derealization, Stress, PTSD, SARS-CoV-2

## Abstract

**Background:**

Dissociative symptoms arise in reaction to significant stress and are common in post-traumatic stress disorder (PTSD).

**Methods:**

We longitudinally investigated the course of dissociative symptoms in psychiatric patients over the first year of the SARS-CoV-2 (severe acute respiratory syndrome coronavirus 2) pandemic situation (04/2020–05/2021). The group was separated into patients with relevant symptoms of PTSD at study start (Group 1: PTSDG) and those without (Group 2: noPTSDG), and we also employed a control group (Group 3: CG). Dissociative symptoms were tracked using the “Fragebogen zu Dissoziativen Symptomen” (FDS), an adapted and extended German version of the “Dissociative Experience Scale” (DES). Datasets of 93 participants were analyzed over up to three time periods. In addition, the regional incidence of positive SARS-CoV-2 tests (7di) and the intensity of non-pharmacological interventions (NPI) were included in multilevel linear models.

**Results:**

Patients reported stronger dissociative symptoms in the PTSDG than in the noPTSDG, while the CG showed the lowest symptom intensity. Importantly, regarding the course of symptoms over time, while public counts of positive SARS-CoV-2 tests had no influence, dissociative symptom severity was significantly impacted by the intensity of non-pharmacological interventions, particularly in the PTSDG and regarding depersonalization and derealization.

**Conclusion:**

Patients with relevant symptoms of PTSD at study start suffered from intense dissociative symptoms over the first year of the SARS-CoV-2 pandemic situation, driven by non-pharmacological interventions. In a milder way, other psychiatric patients were also affected. The results underline the responsivity of these patient groups regarding social and political stress.

**Supplementary Information:**

The online version contains supplementary material available at 10.1186/s40479-026-00363-1.

## Background

This study investigates the development of dissociative symptoms during the first year of the SARS-CoV-2 pandemic situation in psychiatric patients in the German federal state of North Rhine-Westphalia, at the University Hospital Bonn. Hereby, an emphasis lies on patients with symptoms of posttraumatic stress disorder (PTSD) at study start. The SARS-CoV-2 pandemic situation, which lasted for several years, had serious consequences for the global population. These consequences were not limited to the effect of the disease “Covid 19”, caused by SARS-CoV-2, but also encompass specific social and political circumstances. After the declaration of a global pandemic by the WHO (World Health Organization) on March 11, 2020, numerous countries had implemented so-called “non-pharmaceutical Interventions” (NPI) in order to contain the spread of the virus. These included quarantine mandates in the event of infection or contact with an infected person, as well as other restrictions of public and private life [1]. Individuals with pre-existing mental health problems were found to be particularly vulnerable, and a deterioration of their psychiatric symptoms during the pandemic situation was reported in previous investigations [2, 3]. A Brazilian study found that immediately after “social distancing” measures came into force, mental health started to decline in the general population [4]. Other studies revealed that individuals with pre-pandemic psychiatric disorders were specifically at risk, as they showed a low ability to adapt to the changes in everyday life during social restrictions [5, 6].

Regarding psychiatric symptoms that were changing during the course of the pandemic situation, a majority of studies focussed on the evaluation of depressed mood and anxiety [7], while, to the knowledge of the authors, no longitudinal study was published tracking the course of dissociative symptoms in psychiatric patients. Pathological dissociation is primarily understood as a defence mechanism in the context of traumatic experiences or stressful events [8], and facilitates the separation of various mental processes and contents [9]. Dissociative symptoms arise in connection to stressful events and can be assessed with self-report questionnaires, like the DES (Dissociative Experience Scale) [10]. Among specific dissociative symptoms, depersonalisation/derealization (DP/DR) encompass feelings of ‘unreality’ and include emotional numbing, heightened self-observation, changes in body experience, distortions in the experience of time and space, altered sense of agency, feelings of having an empty mind and an inability to focus and sustain attention [11]. In recent time, a growing body of literature revealed new insights into neurobiological correlates of dissociation as a multidimensional and transdiagnostic phenomenon, including alterations in the prefrontal cortex, disconnectivity of the limbic system, hippocampal changes and inflammatory mechanisms [12]. For example, regarding DP/DR, increased prefrontal activation as well as reduced activation in insula/limbic areas is implied [13].

Dissociative symptoms are a common feature in many psychiatric disorders, particularly in borderline personality disorder and post-traumatic stress disorder (PTSD) [14, 15]. Furthermore, dissociation often represents an underrecognized clinical dimension, with potential implications for symptom severity, affective vulnerability, and therapeutic trajectories. Emerging evidence suggests that dissociative symptoms tend to be associated with greater depressive severity and more complex clinical courses, reinforcing the importance of their early identification within psychiatric settings [16]. Elevated states of DP/DR [17], as well as changes in the experience of time [18, 19], were already reported during the SARS-CoV-2 pandemic situation in population-based surveys, yet only one (qualitative) study also assessed patients with pre-existing mental health conditions, some of whom were describing a “sudden loss of a sense of normality” [20].

## Methods

Here, we conducted a longitudinal study to investigate dissociative symptoms over the first year of the Sars-CoV-2 pandemic situation, particularly in patients with PTSD symptoms, and shed light on the contributing roles of childhood trauma, pandemic severity as well as socio-political changes. Psychiatric patients with and without self-reported symptoms of PTSD at study start, as well as control subjects with a self-reported absence of mental disorders, were examined for dissociative symptoms in the context of the SARS-CoV-2 pandemic situation over three time periods (T0, T1, T2), from April 2020 to May 2021. Furthermore, the regional incidence of positive SARS-CoV-2 tests (7-day-incidence, 7di) as well as the local intensity of non-pharmacological interventions (NPI) were assessed as possible contributing factors to acute dissociative symptoms. Participants did not receive any financial or other compensation.

### Measures

#### Essen Trauma Inventory (ETI)

The German self-assessment questionnaire of the Essen Trauma Inventory was used to assess PTSD symptoms at study entry. The instrument is related to the DSM-IV concept of PTSD and is divided into five parts: trauma checklist, inquiry about the worst event, actual post-traumatic symptoms, temporal classification of symptoms and symptom-related restrictions in everyday life. The authors of the questionnaire indicate good to very good values for internal consistency (Cronbach’s alpha) of the scales between 0.82 and 0.95, as well as for the test-retest reliability [21].

For this study, we were particularly interested in the assessment of post-traumatic *symptoms* (intrusions, avoidance behaviour, hyperarousal), aggregated as “ETI-PTSD” value. Items questioning symptom frequency regarding the past month are presented on a 0–3 scale (0 = not at all, 1 = rarely, 2 = often, 3 = very often), with 5 items focussing on intrusions (0–15 points), 7 items on avoidance behaviour (0–21 points) and 5 items on hyperarousal (0–15 points), resulting in a range of 0–51 points for ETI-PTSD. The authors of the questionnaire define the range of 0–16 points as an irrelevant intensity of PTSD symptoms, corresponding to “no PTSD”, while the range from 17 to 51 points defines relevant, at least partial PTSD symptoms. We applied this cut-off to clearly define a group of patients without *any* relevant PTSD symptoms (“noPTSDG”, 0–16 points) from a group of patients with relevant symptoms (“PTSDG”, 17–51 points), fully aware that this does not qualify for a full diagnosis of a PTSD.

#### Childhood Trauma Questionnaire (CTQ)

The Childhood Trauma Questionnaire (German version) was additionally applied to closer assess interpersonal trauma experiences up to the age of 21 years. The 28 item retrospective self-report instrument measures physical and emotional abuse, emotional and physical neglect as well as sexual abuse on a scale of 5 to 25 points per category. The CTQ total score adds up all categories, resulting in a possible range of 25 points (no/minimal trauma load) to 125 points (extreme/maximum trauma load). For the individual scales, the authors provide reliability coefficients (Cronbach’s alpha) between 0.66 (physical neglect) and 0.92 (sexual abuse) [22]. 

#### Dissociative Experience Scale (FDS, German adapted and extended version)

**Fig. 1:**
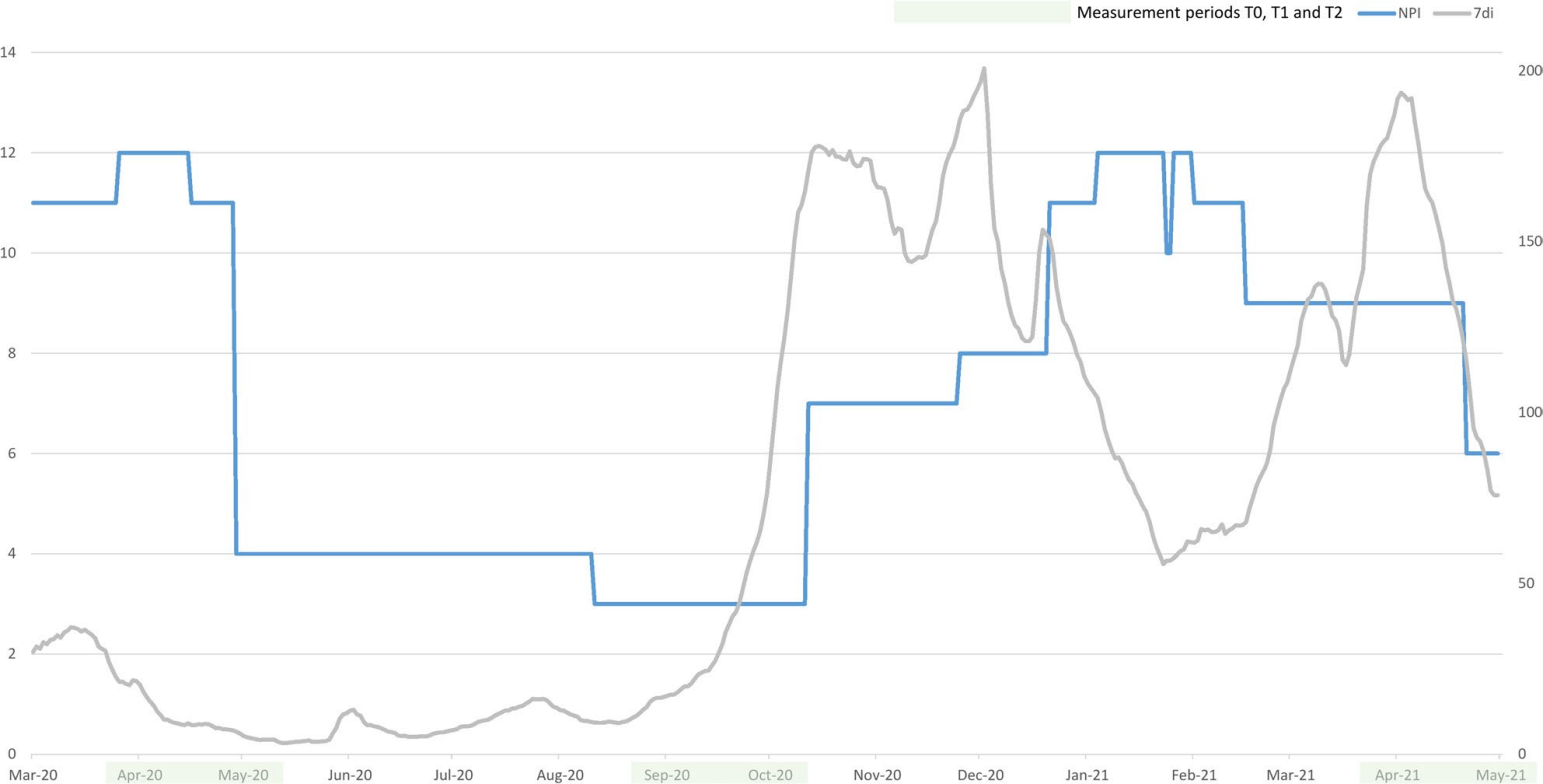
Pandemic situation: Course of NPI-score (blue, left scale [points]) and 7di-score (grey, right scale [positive tests/100.000 people]) over time during the first year of the SARS-CoV-2 pandemic situation in the German state of North-Rhine Westphalia (green: measurement periods T0, T1 and T2)

To measure dissociative symptoms, the third adapted and extended German version of the dissociative experience scale (DES) [10] was used, comprising a total of 44 items (“Fragebogen zu Dissoziativen Symptomen, FDS”) [23]. The instrument is aligned to ICD-10 and DSM-5 concepts of dissociation and allows a broad dimensional assessment, primarily suited for trait diagnostics. To make it useful for measuring longitudinal changes over several months, we added instructions at T1 and T2 to limit the retrospective period of reference (T1: cut-off date 20/05/2020 – significant drop of NPI, T2: cut-off date 01/01/2021 – start of SARS-CoV-II vaccination in Germany and high level of NPI, see Fig. 1). A similar concept underlies the FDS-20, a brief version of the FDS-44 with a satisfactory test-retest-stability, which was shown to be applicable for longitudinal studies. However, the FDS-44 provides a much more comprehensive assessment according to ICD-10 criteria, and the FDS-20 is aimed to a retrospective period of only two weeks [24].The following subscales were assessed in the FDS-44: amnesia, absorption, depersonalization/derealization and conversion. Adding up to an overall score, higher values represent stronger dissociative symptoms. Amnesia encompasses symptoms of autobiographical memory, absorption measures the focus of attention, depersonalization/derealization addresses dissociative changes in perception, which refer to the feeling of being separated from one’s own body or from the environment, and conversion lists symptoms regarding loss of voluntary motor skills and other body-related symptoms. The test-retest reliability of the questionnaire is described as satisfactory to good in various samples, and a very high internal consistency is reported (Cronbach’s alpha = 0.95) [23]. Additionally, also the FDS taxon score was computed, a subscale of 8 items indicating severe forms of dissociation [25]. 

#### Regional incidence of positive SARS-CoV-2 tests (7-day incidence, 7di)

For the state of North Rhine-Westphalia, the Ministry of Labour, Health and Social Affairs published the official, daily updated numbers of positive SARS-CoV-2 PCR tests that were obtained over the course of seven days [26]. These numbers were the source for omnipresent media broadcasts and were mostly presented as daily incidence of COVID-19 cases in North Rhine-Westphalia (see Fig. 1).

#### Score of non-pharmacological interventions (NPI-score)

In order to comprehensibly assess the intensity of non-pharmacological measures to contain viral transmission, a simple score was developed based on the specific regional legislation of the state of North Rhine-Westphalia, taking into account so-called “protection regulations” and “care regulations” [27]. At T0, T1 and T2, the measures were quantified based on the categories “mask mandates”, “general contact restrictions”, “schools”, “gastronomy and retail”, “events”, “health system and social participation” as well as “hotels and cultural institutions”. For each topic, the severity of the measures was rated on a scale of 0 to 2, resulting in a possible total range from 0 to 14 points (see Table 1; Fig. 1). Our regionally specific NPI score was later compared to the Oxford-Stringency Index (OSI) [28], that was developed to compare NPI globally between different countries, and a strong positive correlation (Pearson) was found with the overall OSI score for Germany (*r* = 0.85, *p* <0.001).

**Table 1:** Non-pharmacological intervention score (NPI-score)

Category	NPI intensity		
	0	1	2
Mask mandates	none	Mouth and nose covering	Medical mask/ FFP2/ F95 mask
Contact restrictions	none	max. 5 individuals or 10 individuals from 2 households	max. 2 individuals
Schools	Open (in-person classes)	Hybrid learning	Closed (online classes)
Gastronomy and retail	Open (7 individuals per m²)	Gastronomy closed, retail open	Gastronomy and retail closed (except essential services)
Events	No large-scale events (e.g. fairs), no max. attendance limit but prescribed hygiene concept	Middle-sized events partially prohibited (e.g. concerts), max. attendance limits for allowed events	Events, gatherings, celebrations almost completely prohibited
Healthcare system and social participation	Day-structuring facilities for integration assistance (workshops, day centers) open, hospital visits allowed	Day-structuring facilities open if hygiene requirements are met, hospital visits restricted	Day-structuring facilities closed, hospital visits almost completely prohibited
Hotels and cultural institutions	Hotels open, museums etc. open	Hotels closed, museums etc. open	Hotels, museums, etc. almost completely closed

### Data collecting and processing

Data sets from *N* = 115 individuals were initially obtained. Inclusion criteria were a minimum age of 18 years and a place of residence or work in the German state of North Rhine-Westphalia. Patients (in- and outpatients, as well as daycare-patients) were recruited in the Department of Psychiatry and Psychotherapy (PSY), and to a much smaller extent in the Department of Psychosomatic Medicine and Psychotherapy (PSO) at the University Hospital Bonn (UKB). A convenience sample of control subjects was recruited among hospital staff and their acquaintances.

A number of *n* = 93 datasets were finally analysed (32 male, 61 female, age M = 40.03, SD = 13.52 Y), *n* = 69 from the patient group (PG: 25 male, 44 female, age M = 40.49, SD = 13.89 Y, 62 PSY, 7 PSO) and *n* = 24 from the control group (CG: 7 male, 17 female, age M = 38.71, SD = 12.60 Y). The PG was further divided into patients with relevant self-reported symptoms of PTSD *n* = 41, 15 male, 26 female, age M = 41.49, SD = 14.14 Y (PTSDG) and those without, *n* = 28, 10 male, 18 female, age M = 39.09, SD = 13.63 Y (noPTSDG). Regarding clinical diagnoses (independent from the study), the most frequent primary diagnosis was ICD-10 F33.2 (recurrent depressive disorder, current episode severe without psychotic symptoms), assigned to *n* = 15 patients in the PTSDG and *n* = 8 in the noPTSDG. Overall, borderline personality disorder, characterized by high levels of dissociation, was assigned to *n* = 4 patients in the PTSDG and *n* = 1 patient in the noPTSDG while PTSD was assigned to 8 Patients in the PTSDG and 2 patients in the noPTSDG (see full list of primary and secondary psychiatric diagnoses in supplementary Table S1).

The initial examination period (T0) was undertaken during the first so-called social “lockdown” and covered the period from April 15th to May 17th, 2020. The second examination period (T1) took place from September 10th to October 25th 2020, after schools reopened with face-to-face teaching, until around the start of the 2020/2021 flu season. The third and final examination period (T2) was carried out from April 30th to May 21st, 2021, one year after T0 (see Fig. 1).

### Data analysis

Statistical analyses were performed with SPSS 29 software (IBM Corp., Armonk, NY, USA). Group data were compared at baseline using the Pearson chi square test and analyses of variance for independent samples. Longitudinal data were statistically evaluated using multilevel linear models, a robust method for multivariate analyses with incomplete follow-up data [29]. Initially, the influence of group, NPI-score and 7di-score on the FDS total score and its sub-scores were analysed. Periods of measurements (T0, T1, T2) are reflected in the NPI-score and the 7di-score. Additional models were created to calculate specific interaction effects for individual (sub-) groups. All models were run using robust covariance estimation to address variation. The significance level was set to α = .05.

## Results

Overall, data from *n* = 93 participants (T0) were analysed, with no significant differences between PTSDG, noPTSDG and CG regarding age (F = 0.423, *p* = .656) or sex (χ² = 0.399, *p* = .819). In the longitudinal course of the study, *n* = 41 took part at measurement period T1 and *n* = 28 at T2.

### Descriptive statistics

#### ETI and subgroup assignment

A total of 85 patients and 30 control subjects were eligible. Five participants were excluded due to incomplete / corrupted data (4 patients, 1 control subject), one participant in the PG aborted the study, four participants could not be clearly assigned to a specific study group and nine participants were excluded due to not living or working in the German state of North Rhine-Westphalia (8 patients, 1 control subject). In the CG, three individuals were excluded due to relevant posttraumatic symptoms, leaving *n* = 69 patients and *n* = 24 control participants for further analysis. The ETI was completed at study entry (T0). In the overall patient group (*n* = 69), according to the ETI criteria, the self-report suggested relevant posttraumatic symptoms (ETI-PTSD = 17–51 points) in *n* = 41 patients (subgroup “PTSDG”, M = 31.59, SD = 7.83), while *n* = 28 patients only reached 0–16 points, resulting in the subgroup “noPTSDG” (M = 16, SD = 7.18). In the CG, ETI PTSD reached a maximum of 13 points (M = 4.21, SD = 4.58). Patients lost to follow up (LFU) did not differ significantly from those who ended the study (notLFU) regarding PTSD symptoms in the ETI-PTSD score, neither in the PTSDG (LFU: M = 31.57, SD = 8.39; notLFU: M = 31.00, SD = 6.75; t = 0.22, *p* = 0.831) nor in the noPTSDG (LFU: M = 6.86, SD = 5.71; notLFU: M = 8.14, SD = 5.01; t = -0.53, *p* = 0.601) or in the CG (LFU: M = 4.40, SD = 4.73; notLFU: M = 3.89, SD = 4.57; t = 0.26, *p* = 0.798). As the worst traumatic experience (in person or as a witness), participants in the PTSDG reported sexual assault by a stranger in adulthood (2.44%, *n* = 1), sexual abuse by a stranger in childhood (4.88%, *n* = 2), sexual abuse by a family member/acquintance in childhood (4.88%, *n* = 2), sexual assault by a family member/acquintance in adulthood (4.88%, *n* = 2), neglect/estrepement (9.76%, *n* = 4), life threatening illness (14.63%, *n* = 6), death of a close person or family member (14.63%, *n* = 6) and other traumatic events (29%, *n* = 12). The other traumatic events comprised stressful experiences in a partnership (including separation), divorce of parents, mobbing, and the onset of a depressive episode. In 15% (*n* = 6), no worst event was indicated, while those patients reported between 2 and 13 different traumatic experiences. Regarding duration of PTSD symptoms, 7.32% (*n* = 3) reported <1month, 19.51% (*n* = 8) 1–3 months and a majority of 73.17% (*n* = 30) indicated > 3 months.

#### CTQ

The CTQ was conducted once, during the first study period (T0). Due to missig values, we were able to calculate the CTQ total score for 89 participants (40 PTSDG, 25 noPTSDG, 24 CG). As expected, results showed the highest trauma load in the PTSDG (M = 52.38, SD = 19.12) followed by the noPTSDG (M = 39.76, SD = 9.78) and finally the CG (M = 32.92, SD = 10.01). A one-way ANOVA revealed a statistically significant group difference (F = 14.12, *p* < .001, eta² = 0.25).

#### FDS

For the FDS (data from *n* = 90 participants available), total scores and sub-scores were compared using multilevel linear models. One patient was excluded as an extreme outlier.

##### FDS total and taxon score

**Fig. 2:**
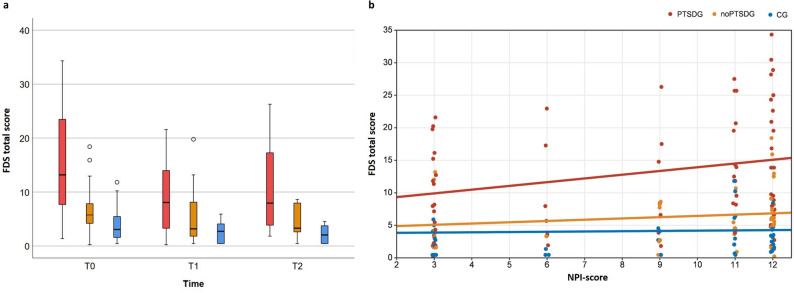
FDS total score and dependence from NPI score. **a** (left) Boxplots show FDS total scores for every group and time period. **b** (right) Graph shows increase of FDS total score depending on NPI-score for every group

Participants of the CG had the lowest dissociation values during all study periods, while the PTSDG consistently showed the highest total (see Fig. 2a) and taxon scores, see Table 2. Regarding the taxon score, no group at no timeperiod reached a mean value above 20 points that might indicate severe dissociation. However, the PTSDG surpassed 5 points at T0 and T2, a value that is only reached by less than 10% of the general population in Germany [25]. 

**Table 2:** FDS total score, sub-scores and taxon-score

		PTSDG	noPTSDG	CG
**FDS total score (M, SD)**				
	T0	14.67, 9.3	6.56, 4.35	3.85, 3.02
	T1	9.37, 6.72	5.82, 6.28	2.65, 2.05
T1 Missing data		20	17	15
	T2	10.67, 8.2	4.79, 3.3	2.19, 1.72
T2 Missing data		28	21	16
**FDS Sub-scores (M, SD)**				
Amnesia	T0	9.39, 11.8	3.79, 4.68	1.72, 2.58
Absorption		23.56, 13.75	14.43, 9.56	8.54, 7.02
DP/DR		12.53, 15.37	4.11, 5.98	1.39, 2.34
Conversion		10.96, 10.72	2.5, 4.01	2.01, 3.27
Amnesia	T1	4.71, 6.55	7.39, 7.82	3.06, 6.5
Absorption		18.58, 12.17	17.05, 19.47	5.11, 3.5
DP/DR		5.43, 6.27	2.95, 5.07	0.74, 1.21
Conversion		7.11, 7.55	0.81, 1.58	3.58, 6.93
T1 Missing data		20	17	15
Amnesia	T2	7.4, 9.03	2.86, 4.06	0.63, 1.34
Absorption		19.18, 13.63	12.68, 7.45	3.28, 2.58
DP/DR		7.44, 10.66	2.02, 3.33	0.21, 0.59
Conversion		9.06, 9.97	1.75, 3.26	1.81, 3.85
T2 Missing data		28	21	16
**FDS Taxon-score (M, SD)**				
	T0	10.54, 9.86	3.06, 4.13	1.25, 1.73
	T1	4.63, 4.31	3.64, 6.19	0.97, 1.63
T1 Missing data		20	17	15
	T2	6.15, 7.15	1.07, 1.52	0.00, 0.00
T2 Missing data		28	21	16

##### FDS total score – multilevel linear model I

An initial multilevel linear model was applied to analyse the influence of group, NPI-score and 7di-score on the FDS total score over time. Here, statistically significant effects for group (*p* < .001) and NPI-score (*p* < .001) emerged. The effect of 7di-score was also significant (*p* = .005), but with a negative correlation. Results reveal that for an increase in NPI-score by one point, the FDS total score increases by an average of 0.35 points. The PTSDG shows an average FDS total score that is 9.50 points higher than in the CG and 7.33 points higher than in the noPTSDG, adjusted for NPI-score and 7di-score, see fixed parameter estimates of the FDS total score, Table 3.

**Table 3:** FDS total score, multilevel linear model I and II

FDS total score	Parameter estimation	95%- CI	*p*-value
**Multilevel linear model I**			
constant term	0.76	-1.08–2.60	-
group (reference = CG)			< .001
PTSDG	9.5	6.76–12.24	
noPTSDG	2.17	0.33–4.01	
NPI-score	0.35	0.20–0.50	< .001
7di-score	-0.02	-0.03 – (-0.01)	.005
**Multilevel linear model II**			
constant term	3.76	2.14–5.39	-
group (reference = CG)			.006
PTSDG	4.43	1.70–7.16	
noPTSDG	0.73	-2.09–3.55	
NPI-score	0.04	-0.08–0.16	< .001
group*NPI-score (reference = CG)			< .001
PTSDG * NPI-score	0.53	0.27–0.79	
noPTSDG * NPI-score	0.15	-0.05–0.36	
7di-score	-0.02	-0.03–0.01	.004

##### FDS total score – multilevel linear model II

Since the NPI-score shows a significant overall effect on the FDS score, another mixed linear model was employed in order to break down this result on the group level, unveiling a significant interaction effect between group and NPI-score (*p* < .001). An increase of the NPI-score by one unit is associated with an increase of the FDS total score by 0.04 points in the CG, by 0.15 points in the noPTSDG, and by 0.53 points in the PTSDG (Table 3: FDS total score, multilevel linear model II, Fig. 2b: Increase of the FDS total score depending on the NPI-score.)

##### FDS sub-scores

For the FDS sub-scores “absorption”, “conversion” and “depersonalization/derealization”, the values of the PTSDG exceeded those of the other groups at any times. This also holds mostly true for the sub-score “amnesia”: here however, at T1, the noPTSDG showed higher values than the PTSDG, see Table 2.

##### FDS sub-scores: multilevel linear models I

The amnesia sub-score showed a statistically significant group effect (*p* < .001), but neither a statistically significant effect for the NPI-score (*p* = .476), nor for the 7di-score (*p* = .272). The absorption sub-score revealed a statistically significant effect for group (*p* < .001) and a statistically significant positive effect for NPI-score (*p* = .015), as well as a statistically significant negative effect for 7di-score (*p* = .026). For the sub-score depersonalization and derealization, there was a statistically significant effect for group (*p* < .001) and a statistically significant positive effect for NPI-score (*p* = .001), but none for 7di-score (*p* = .137). For the sub-score conversion, only a statistically significant effect for group (*p* < .001) was shown, but no effect for NPI-score (*p* = .111) or 7di-score (*p*= .320), see Table 4.

**Table 4:** FDS sub-scores, multilevel linear model I and II (for DP/DR)

FDS sub-scores		Parameter estimation	95%-CI	*p*-value
**Multilevel linear model I**				
Amnesia	constant term	0.99	-3.15–5.13	-
	group (reference = CG)			< .001
	PTSDG	5.91	3.08–8.73	
	noPTSDG	2.5	0.40–4.63	
	NPI-score	0.13	-0.23–0.50	.476
	7di-score	-0.01	-0.03–0.01	.272
Absorption	constant term	5.56	1.99–9.12	-
	group (reference = CG)			< .001
	PTSDG	14.22	9.72–18.73	
	noPTSDG	5.83	1.52–10.15	
	NPI-score	0.32	0.06–0.58	.015
	7di-score	-0.03	-0.05–0.00	.026
DP/DR	constant term	-1.25	-3.10–0.60	-
	group (reference = CG)			< .001
	PTSDG	10.16	5.54–14.78	
	noPTSDG	2.78	0.43–5.12	
	NPI-score	0.27	0.11–0.43	.001
	7di-score	-0.01	-0.02–0.00	.137
Conversion	constant term	0.81	-2.03–3.64	-
	group (reference = CG)			< .001
	PTSDG	7.71	4.39–11.03	
	noPTSDG	-0.3	-2.14–1.53	
	NPI-score	0.18	-0.04–0.40	.111
	7di-score	-0.01	-0.02–0.01	.320
**Multilevel linear model II**				
DP/DR	constant term	0.71	-0.74–2.15	-
	group (reference = CG)			.009
	PTSDG	6.1	1.61–10.61	
	noPTSDG	3.49	-0.19–7.17	
	NPI-score	0.07	-0.06–0.20	
	group *NPI-score (reference = CG)			.008
	PTSDG *NPI-score	0.43	0.14–0.71	
	noPTSDG *NPI-score	-0.06	-0.32–0.19	
	7di-score	-0.01	-0.12–0.00	.116

##### FDS sub-scores: multilevel linear models II

For sub-scores that were significantly influenced by either 7di- or NPI-score, additional multilevel linear models where obtained to investigate differential group effects.

**Fig. 3:**
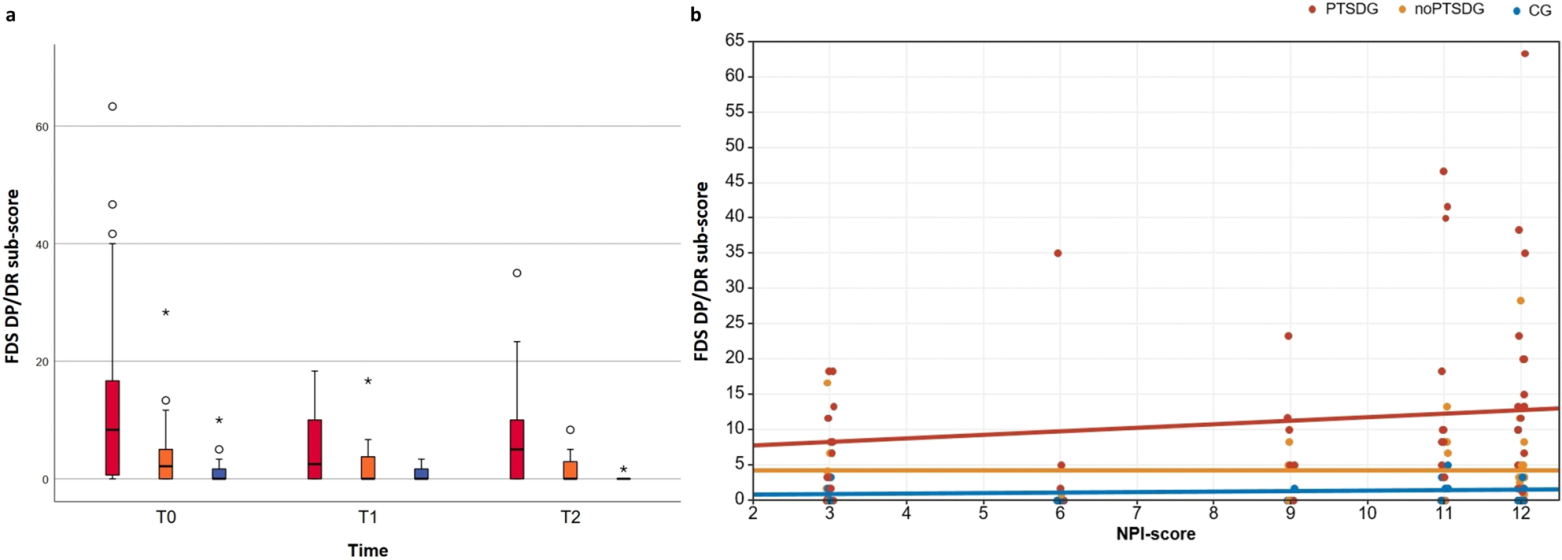
FDS DP/DR sub-score and dependence from NPI score. **a** (left) Boxplots show scores for every group and time period. **b** (right) FDS total score depending on NPI score. Graph shows increase of DP/DR sub-score depending on NPI-score for every group

For the sub-score “absorption”, NPI-score (*p* = .313) and 7di-score (*p* = .968) did not differentially affect specific study groups. However, for the sub-score DP/DR, a statistically significant group interaction was found for NPI-score (*p* = .008), see Table 4; Fig. 3 (Fig. 3a: FDS DP/DR sub-score, Fig. 3b: Increase of the FDS sub-score “DP/DR” depending on the NPI score.)

### Summary of results

The PTSDG showed the greatest scores of dissociative symptoms compared to the noPTSDG and the CG. The dynamics of the FDS total score over time were hereby driven by changes in the NPI-score: The higher the NPI-score, the greater the severity of the dissociative symptoms, particularly in the PTSDG. In contrast, the 7di-score did not show any positive effect on the FDS total score. The sub-score “depersonalization and derealization” reflected the FDS total score almost identically, also resulting in a statistically significant interaction effect of group and NPI score. For “absorption”, there was a statistically significant effect of the NPI-score, but no significant interaction with individual groups. The “amnesia” and “conversion” sub-scores showed changes over the course of the study, yet independent of the NPI-score.

## Discussion

Our results are in line with several studies that demonstrated a higher overall vulnerability for psychiatric patients [5, 30], including those with PTSD [31], in regard to the SARS-CoV-2 pandemic situation. At an early stage of the pandemic, a study from Bavaria showed that individuals with severe mental illness were more likely to state that the situation had a negative impact on their psychiatric condition [32]. Furthermore, investigations in the German state of Lower Saxony described that during the pandemic situation individuals with psychiatric disorders were prone towards a deterioration of their symptoms [3], and that the perception of distress was significantly increased in spring and declined towards autumn 2020 [33]. In addition, a North American group analysed healthy participants and psychiatric patients in the USA in April/May 2020, and reported that non-pharmacological measures significantly increased psychological distress, especially among individuals with pre-existing mental disorders [30].

Our study extends these results in focussing on dissociative symptoms. We were able to show an interconnection of dissociative symptom intensity and the extent of non-pharmacological interventions in all three groups, using mixed linear models. While the NPI had the least impact on dissociative symptoms in the CG, the influence was slightly higher in the noPTSDG and considerably elevated in the PTSDG. The 7di-score showed no or even negative correlations, which are best interpreted as statistical artefacts. In conclusion, strict NPI (e.g. at T0) had a clearly greater influence on the intensity of dissociative symptoms than high incidences of positive PCR tests, as were observed at T1 and T2. It needs to be noted, however, that the statistical influence of the NPI on dissociative symptoms should not be conflated with causality, keeping in mind the observational nature of the study and the lack of control for alternative explanations.

A further refinement of the results considers the FDS subscales, assessing the individual dissociative symptom profile [23], particularly regarding “depersonalization and derealization” (DP/DR). In this sub-score, the FDS captures “the experience of strangeness, unreality, being separated or detached from one’s own self, the feelings of alienation and the experience of unreality [related] to the immediate environment” [23]. In our study, the DP/DR sub-score shows a significant interaction result over time with the NPI-score, predominantly in the PTSDG. The feeling of DP/DR seems to be particularly pronounced in this group at T0, which suggests that the pandemic situation, and in particular the NPI, were experienced by these patients as somehow “unreal”. It is reported that, in general, individuals who suffer from experiences of DP/DR perceive themselves as helpless, hopeless and socially isolated [34]. Attention has already been drawn to a lack of adaptation to the changed living conditions among individuals with previous mental illnesses under pandemic stress [5], resulting in a reduced “sense of normality”, as reported in a qualitative study [20]. It appears plausible that the feeling of DP/DR in patients with potential stress related disorders, like PTSD, could be seen as an expression of perceived feelings of threat in response to the profound changes in the immediate living conditions. Similarly, a cross-sectional online study from Hungary found that perceived stress moderately correlated with dissociative phenomena during the pandemic situation [35]. In this regard, contemporary models interpret dissociation not only as a trauma-related phenomenon, but also as a marker of greater psychopathological complexity [36], associated with affective dysregulation, depressive severity, and increased need for clinical intervention [37].

As early as 2020, the concept of a state of emergency as a “new normal” was used by high ranking politicians like the then minister of finance and later chancellor of Germany, Olaf Scholz, and was broadcasted over various newspapers and tv channels, e.g. “ARD Tagesschau” [38] and “Die Welt” [39]. This “new reality” implied a distinction from a “previous reality”, which can explain a feeling of “de-realization”. Patients with stress related disorders presumably perceived this change in a particular serious way, because previous life events have taught them to detect potential dangers in an early stage, which is reflected in the PTSD criterion of “hypervigilance” [21]. The affected patients may have reacted so strongly to the new pandemic situation because they knew that realities, including interpersonal realities, may change unexpectedly, and seemingly peaceful situations can quickly turn into threatening scenarios. Individuals with traumatization, particularly in childhood, are also considered to be more vulnerable to social withdrawal and to the development of PTSD and dissociative symptoms in adulthood [40, 41]. Furthermore, childhood trauma is associated with structural and connectivity changes in limbic areas and may increase the risk for later dissociative symptoms under stressfull conditions [42–44].

Dissociation can be interpreted as an excessive, partially dysfunctional protective mechanism, which is often based on traumatic experiences and is used to combat perceived threatening stress [14], just like during the pandemic situation [32]. This behaviour may be facilitated via stimulus generalization, a phenomenon observed in patients with PTSD symptoms, leading to the identification of situational triggers as trauma-associated, which might be very dissimilar to the original traumatic situation [45].

Our study also comes along with limitations. Initially, we intended to survey the test subjects in spring 2020, and a second time after the end of the pandemic situation, which we initially expected for summer 2020. Since the pandemic situation lasted longer, the study was extended with another survey in spring 2021, one year after study start. We did not proceed with the study even longer, because the NPI became more and more nuanced in 2021. As a simple measure, the mean number of pages of the continuously updated “Corona Protection Bill” of the state of North Rhine-Westphalia increased substantially over the course of the study (T0 = 11.60 pages, T1 = 20.40 pages and T2 = 24.75 pages) [27]. Furthermore, the number of positive PCR tests reached 421/100,000 residents as of May 17, 2022 (two years after T0), showing a significant increase compared to our survey periods (T0: 16.98/100,000 residents, T1: 21.21/ 100,000 residents and T2 127.69/ 100,00 residents) [26]. However, these high incidence figures were not reflected in a further tightening of the NPI, so that an increasing decoupling between detection rates of Sars-CoV-2 and non-pharmacological interventions was observed. Vaccinations against COVID-19 in Germany started in late December 2020 [46]. From spring to summer of 2021, this medical intervention was available to the general population across the board. In addition, from March 2021 it was increasingly easy to test oneself for a contact with SARS-CoV-2 using rapid antigen tests. Altogether, it can be concluded that spring 2021 was the beginning of a new phase of the pandemic situation, which was no longer followed up upon in this study. After the end of data collection at T2, potential results of further surveys would have been increasingly difficult to interpret and lesser comparable to the initial events.

The study had to start quickly in order to examine the early stages of the situation. Here, a face-to-face study recruitment was severely limited due to strict hygiene measures and other official restrictions on everyday life. This lead to a comparably low number of datasets, which even decreased over the course of survey periods, presumably due to a declining interest in further participation. Furthermore, diagnostics rely completely on self-report measures, considerably affecting the conclusions that can be drawn from the study. Additionally, a potential overlap between temporal effects, pandemic progression, and NPI-related effects might be considered and contribute to a more nuanced interpretation of the results. 

Regarding the high amount of participants lost to follow up, a reduction of information on within group variance for T1 and T2 is clearly limiting the interpretation of the results. However, by applying linear multilevel models, we were able to include all available data points. Although we are unable to know the specific reasons for discontinuation, a comparison of ETI-PTSD scores at T0 of participants lost to follow up with those who continued the study revealed no significant differences, cautiously speaking against systematic dropouts and in favour of a missing at random assumption, implying a relative robustness of the analyses. 

In the present study, patients were allocated to subgroups with regard to PTSD symptoms at study entry, using the ETI-PTSD self-assessment score. Hereby, we decided against a full diagnostic process for PTSD, which would have required a structural diagnostic interview such as the CAPS [47]. Due to lengthy direct conversations that would have been necessary for such an interview, we abstained from this procedure according to hygiene recommendations. Likewise, the exclusion of mental illnesses in the control group was also assessed via self report and not carried out using a scientifically established protocol, such as the SCID [48].

The CTQ showed that the participants in the PTSDG had suffered significantly more childhood traumas than those in the noPTSDG (see above), hereby the discriminating effect of a positive self report for relevant PTSD symptoms might be conflated. Furthermore, the relevance of changed media consumption during the pandemic situation, which was suspected in another study with regard to the development of DP/DR symptoms [17], was not examined here.

Reference to mortality rates in connection with SARS-CoV-2 was deliberately omitted, as the published figures did not indicate whether a person died “from” or “with” the virus. In this regard, a German study states SARS-CoV-2 as the cause of death for around 85% of the reported SARS-CoV-2 deaths [49], an earlier study from Hamburg, however, reported 80 consecutive autopsies of SARS-CoV-2 deaths, of which 78 cases had relevant comorbidities as further possible causes of death [50]. Elsewhere, concerns were raised that estimates of death rates cannot be interpreted independently of the indirect effects of the pandemic, such as the impact of the NPI [51]. Overall, the death rates appear significantly less meaningful than the incidence numbers used in this study (7di), which are considered reliable for the detection of SARS-CoV-2 mRNA (messenger ribonucleic acid) material.

## Conclusion

Taken together, our study was able to show a considerable responsivity of psychiatric patients, particular those with relevant PTSD symptoms at study entry, regarding a modulation of dissociative symptoms under the multifaceted stress during the SARS-CoV-2 pandemic situation. Hereby, restrictions of everyday living conditions played an important role as an aggravating factor. The authors recommend a careful adjustment of non-pharmaceutical interventions for future scenarios, keeping in mind the need to protect vulnerable populations like psychiatric patients, particularly those with potential pre-existing stress disorders. Furthermore, systematic screenig for dissociative symptoms in mental health services is advised, considering that these manifestations are frequently underdiagnosed, despite their potential impact on psychopathological severity, treatment response, and patterns of healthcare utilization.

## Supplementary Information

Below is the link to the electronic supplementary material.

Supplementary Material 1

## Data Availability

Underlying data of the study are available in the bonndata repository:10.60507/FK2/X3YVXM.

## Abbreviations

7di: Incidence of positive SARS-CoV-2 tests over 7 days
CAPS: Clinician-Administered PTSD Scale
CG: Control group
COVID-19: Coronavirus disease 2019
CTQ: Childhood Trauma Questionnaire
DES: Dissociative Experience Scale
DP/DR: Depersonalisation/Derealization
DSM: Diagnostic and statistical manual of mental disorders
ETI: Essen Trauma Inventory
FDS: Fragebogen zu dissoziativen Symptomen
ICD: International classification of diseases and related health problems
M: Mean value
mRNA: Messenger ribonucleic acid
noPTSDG: Patient group with no relevant symptoms of PTSD at study start
NPI: Intensity of non-pharmacological interventions
PTSD: Post-traumatic stress disorder
PTSDG: Patient group with relevant symptoms of PTSD at study start
SARS-CoV-2: Severe acute respiratory syndrome coronavirus 2
SCID: Structured Clinical Interview for DSM
SD: Standard deviation
SPSS: Statistical package for the social sciences
T0: Initial examination period (April 15th to May 17th, 2020)
T1: Second examination period (September 10th to October 25th, 2020)
T2: Third and final examination period (April 30th to May 21st, 2021)
UKB: University Hospital Bonn
WHO: World Health Organization
