# Supplementary material for: Dissociative symptoms during the SARS-CoV-2 pandemic situation in mental health patients
Source: Borderline Personal Disord Emot Dysregul. 2026 Jul 27;13:22. doi: 10.1186/s40479-026-00363-1 (PMC13403852; doi:10.1186/s40479-026-00363-1)
Supplement: Supplementary file 1 — Supplementary Material 1 [file 40479_2026_363_MOESM1_ESM.pdf]

Supplementary Material, Table S1: Primary and Secondary Diagnoses of PTSDG and noPTSDG

**Primary Diagnoses (ICD-10)**

| PTSDG (n=41) | Frequency | % Patients |
|---|---|---|
| F33.2 | 15 | 36.59 |
| F31.4 | 3 | 7.32 |
| F11.2 | 2 | 4.88 |
| F20.0 | 2 | 4.88 |
| F32.1 | 2 | 4.88 |
| F43.1 | 2 | 4.88 |
| F60.31 | 2 | 4.88 |
| F10.2 | 1 | 2.44 |
| F23.3 | 1 | 2.44 |
| F31.3 | 1 | 2.44 |
| F31.6 | 1 | 2.44 |
| F31.7 | 1 | 2.44 |
| F32.2 | 1 | 2.44 |
| F32.8 | 1 | 2.44 |
| F41.2 | 1 | 2.44 |
| F42.1 | 1 | 2.44 |
| F43.2 | 1 | 2.44 |
| F50.0 | 1 | 2.44 |
| F60.0 | 1 | 2.44 |
| F90.0 | 1 | 2.44 |
| **Primary diagnoses in PTSDG** | **41** | |

| noPTSDG (n=28) | Frequency | % Patients |
|---|---|---|
| F33.2 | 8 | 28.57 |
| F90.0 | 3 | 10.71 |
| F42.2 | 3 | 10.71 |
| F11.2 | 2 | 7.14 |
| F31.4 | 2 | 7.14 |
| F20.0 | 2 | 7.14 |
| F63.9 | 1 | 3.57 |
| F50.0 | 1 | 3.57 |
| F43.1 | 1 | 3.57 |
| F42.0 | 1 | 3.57 |
| F33.3 | 1 | 3.57 |
| F33.1 | 1 | 3.57 |
| F33.0 | 1 | 3.57 |
| F25.2 | 1 | 3.57 |
| **Primary diagnoses in no PTSDG** | **28** | |

**Secondary Diagnoses (ICD-10)**

| PTSDG (n=41) | Frequency | % Patients |
|---|---|---|
| F43.1 | 8 | 19.51 |
| F17.2 | 7 | 17.07 |
| F33.2 | 5 | 12.20 |
| F10.2 | 4 | 9.76 |
| F10.3 | 3 | 7.32 |
| F33.1 | 3 | 7.32 |
| F41.0 | 3 | 7.32 |
| F90.0 | 3 | 7.32 |
| F40.01 | 2 | 4.88 |
| F41.1 | 2 | 4.88 |
| F45.41 | 2 | 4.88 |
| F54 | 2 | 4.88 |
| F60.31 | 2 | 4.88 |
| F61 | 2 | 4.88 |
| F10.1 | 1 | 2.44 |
| F10.4 | 1 | 2.44 |
| F11.3 | 1 | 2.44 |
| F12.2 | 1 | 2.44 |
| F12.3 | 1 | 2.44 |
| F13.3 | 1 | 2.44 |
| F33.3 | 1 | 2.44 |
| F40.1 | 1 | 2.44 |
| F42.0 | 1 | 2.44 |
| F42.2 | 1 | 2.44 |
| F43.0 | 1 | 2.44 |
| F44.5 | 1 | 2.44 |
| F45.0 | 1 | 2.44 |
| F50.4 | 1 | 2.44 |
| F60.30 | 1 | 2.44 |
| F63.8 | 1 | 2.44 |
| F66.02 | 1 | 2.44 |
| **Secondary Diagnoses in PTSDG** | **65** | |
| **Secondary Diagnoses per patient in PTSDG** | **1.59** | |

| noPTSDG (n=28) | Frequency | % Patients |
|---|---|---|
| F10.2 | 2 | 7.14 |
| F10.3 | 2 | 7.14 |
| F11.3 | 2 | 7.14 |
| F13.2 | 2 | 7.14 |
| F17.2 | 2 | 7.14 |
| F33.1 | 2 | 7.14 |
| F41.0 | 2 | 7.14 |
| F41.1 | 2 | 7.14 |
| F10.0 | 1 | 3.57 |
| F12.2 | 1 | 3.57 |
| F12.3 | 1 | 3.57 |
| F13.3 | 1 | 3.57 |
| F19.1 | 1 | 3.57 |
| F20.0 | 1 | 3.57 |
| F33.0 | 1 | 3.57 |
| F33.2 | 1 | 3.57 |
| F332 | 1 | 3.57 |
| F41.2 | 1 | 3.57 |
| F42.2 | 1 | 3.57 |
| F43.1 | 1 | 3.57 |
| F54 | 1 | 3.57 |
| F60.31 | 1 | 3.57 |
| F61 | 1 | 3.57 |
| F63.8 | 1 | 3.57 |
| F66.4 | 1 | 3.57 |
| **Secondary Diagnoses in noPTSDG** | **33** | |
| **Secondary Diagnoses per patient in noPTSDG** | **1.18** | |
